# Recent Advances and Challenges in Nanodelivery Systems for Antimicrobial Peptides (AMPs)

**DOI:** 10.3390/antibiotics10080990

**Published:** 2021-08-16

**Authors:** Ziyan Tang, Quantao Ma, Xiaoling Chen, Tianbao Chen, Yuan Ying, Xinping Xi, Lei Wang, Chengbang Ma, Chris Shaw, Mei Zhou

**Affiliations:** 1 Natural Drug Discovery Group, School of Pharmacy, Queen’s University Belfast, Belfast BT9 7BL, UK; tangziyan1997@163.com (Z.T.); t.chen@qub.ac.uk (T.C.); y.ying@qub.ac.uk (Y.Y.); x.xi@qub.ac.uk (X.X.); l.wang@qub.ac.uk (L.W.); c.ma@qub.ac.uk (C.M.); chris.shaw@qub.ac.uk (C.S.); 2 School of Traditional Chinese Medicine, Beijing University of Chinese Medicine, Beijing 100029, China; maquantao@bucm.edu.cn

**Keywords:** antimicrobial peptides, antibiotics, nanodrug delivery systems, resistance

## Abstract

Antimicrobial peptides (AMPs) can be used as alternative therapeutic agents to traditional antibiotics. These peptides have abundant natural template sources and can be isolated from animals, plants, and microorganisms. They are amphiphilic and mostly net positively charged, and they have a broad-spectrum inhibitory effect on bacteria, fungi, and viruses. AMPs possess significant rapid killing effects and do not interact with specific receptors on bacterial surfaces. As a result, drug resistance is rarely observed with treatments. AMPs, however, have some operational problems, such as a susceptibility to enzymatic (protease) degradation, toxicity in vivo, and unclear pharmacokinetics. However, nanodelivery systems loaded with AMPs provide a safe mechanism of packaging such peptides before they exert their antimicrobial actions, facilitate targeted delivery to the sites of infection, and control the release rate of peptides and reduce their toxic side effects. However, nanodelivery systems using AMPs are at an early stage of development and are still in the laboratory phase of development. There are also some challenges in incorporating AMPs into nanodelivery systems. Herein, an insight into the nanotechnology challenges in delivering AMPs, current advances, and remaining technological challenges are discussed in depth.

## 1. Introduction

### 1.1. The Medical Value of AMPs and Their Mechanisms of Bioactivity

Antimicrobial peptides (AMPs) have significant potential as alternatives to replace conventional antibiotics, as they can more effectively inhibit susceptible pathogens, as well as multidrug-resistant (MDR) bacteria and fungi [1]. The World Health Organization (WHO) estimated that resistance to traditional antibiotics would give rise to the deaths of at least 10 million people by 2050 [2,3]. It is worth looking for novel antibiotics or combining antibiotics with delivery systems to tackle infectious diseases caused by resistant organisms.

AMPs are also called host defensive peptides (HDPs), as they actively participate in host defense mechanisms and display biological activities. They usually contain 11–50 amino acids [4]. Being amphiphilic and mostly positively charged, they exhibit broad-spectrum effects on Gram-negative and Gram-positive bacteria, viruses, and fungi [5]. Furthermore, these peptides exert significant antibacterial effects by disrupting bacterial cell membranes, modulating the immune response, and regulating inflammatory factors [6]. They can also exert a synergistic effect with current antibiotics to enhance antibacterial activity [7].

**Figure 1:**
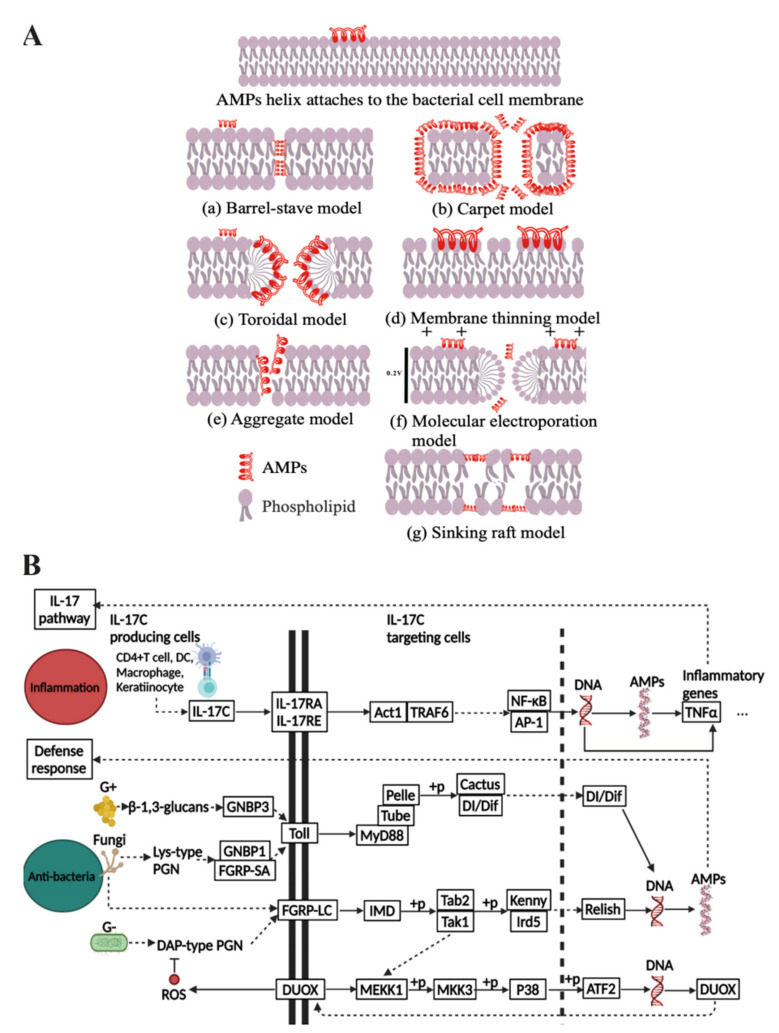
(**A**) Membrane lysis antibacterial mechanisms of AMPs (AMPs in red). (**B**) Putative models of inflammation and antibacterial pathways of AMPs by making use of pathway data from KEGG database [24].

AMP mechanisms of action are divided into membrane lysis, nonmembrane lysis, and immunomodulation [8,9,10]. The interaction between AMPs and the membranes of microorganisms occurs through electrostatic attraction and hydrophobic interaction. These positively charged peptides are electrostatically attracted to the negatively charged components of the microorganisms and then interact with the cell membrane by hydrophobic interaction to disrupt the integrity of the microbial membrane, ultimately leading to cellular necrosis or apoptosis [11]. The membrane lysis mechanism is one of the main reasons for killing microorganisms rapidly and maintaining this without inducing resistance in bacteria. The mechanisms of this common effect can be explained through several models. The lysis process can be detected by oriented circular dichroism (OCD) and nuclear magnetic resonance spectroscopy (NMR), which taken together can explain membranolytic effects by the barrel-stave model, carpet model, membrane thinning model, aggregate model, molecular electroporation model, toroidal model, and sink raft model (Figure 1A) [12,13]. These peptides disrupt microbial membranes by changing the membrane arrangement or the charge on both sides of the membrane in different patterns, and these models are the basis for their expressed biological activity [14,15,16,17,18,19].

Moreover, AMPs present a broad range of other bioactivities such as anti-inflammatory, anticancer, and promoting tissue regeneration and repair [20,21,22]. For these reasons, these active peptides are gaining interest as potential drug candidates for complex diseases and multidrug-resistant pathogen infections [23].

Inflammation, bacterial infection, and cancer cause the production of AMPs, which exert anti-inflammatory and self-defense effects to protect the host body from damage. The KEGG PATHWAY Database showed that the anti-inflammatory and antibacterial signaling pathways of AMPs are well studied (Figure 1B) [24]. Firstly, the inflammatory response causes peptides to act as anti-inflammatories relating to the IL-17 signaling pathway. The IL-17 family signals via correspondent receptors and activates downstream pathways to induce the expression of AMPs. When the human body undergoes an immune response, the T cells can produce interleukins (IL-17C) and then produce Act1 through connecting with specific receptors, such as NF-κB, which is an activator and also thought to be the master mediator in this pathway. It ultimately regulates the production of AMPs through the regulation of DNA and modulation of inflammatory factors. Secondly, in terms of antimicrobial pathways, Gram-positive bacteria give rise to the Toll pathway and cause the production of AMPs for antimicrobial purposes, while Gram-negative bacteria contribute to the IMD pathway, and fungi tend to activate both pathways. In the Toll pathway, microorganisms affect the Dorsal and Dif, which can induce the upregulation of transcription of AMPs through recognition proteins GNBP and FGRP-SA. In the IMD pathway, microorganisms cause the upregulation of transcription of AMPs in the nucleus by recognition protein FGRP-LC, which leads to the production of immunodeficiency (IMD) and regulates Relish through a series of phosphorylation responses. Moreover, the IMD pathway also induces ROS to produce antimicrobial effects. Finally, the anticancer signaling pathway of AMPs has not been fully investigated, but it has been suggested as being related to the CXCR4-Akt pathway, where AMPs inhibit CXCR4 expression, leading to reduced phosphorylation of AKT, causing the accumulation of P21 and inactive forms of CDC2, finally leading to cell cycle arrest in the G2/M phase [25].

### 1.2. The Challenges of AMP Delivery and the Advantages of Combining with Nanosystems

Although many AMPs exhibit antibacterial or anticancer activities, several studies suggested that some problems arise with their traditional delivery. Firstly, it has been reported that the susceptibility of bacteria to cationic peptides is reduced, driven by the production of proteases or by trapping proteins and altering the surface charge of cells through self-modification [26,27]. Secondly, some active peptides are toxic to eukaryotic cells and can cause neurotoxicity, nephrotoxicity, or haemolysis [28]. Thirdly, these peptides are unstable during metabolism in vivo due to their pharmacokinetic properties, high sensitivity to proteases, short half-life, and instability under physiological conditions [29].

AMP delivery using nanodelivery systems provides an effective solution for the problems with these peptides [30]. Firstly, the problem of decreased sensitivity of AMPs can be alleviated by forming a drug delivery system with a nanocarrier, which could enhance the antibacterial effect by acting synergistically with AMPs. It has been reported that silver oxide nanoparticles are a type of special nanocarrier for antibiotics, and silver nanoparticles bound to the peptide Odorranain-A-OA1 were more potent in inhibiting *Escherichia coli* (*E. coli*). This demonstrates that nanoparticles can act synergistically with AMPs to enhance biological activity [31].

Secondly, the combination of AMPs with nanodelivery techniques has become an excellent strategy to decrease the cytotoxicity of AMPs [30]. On the one hand, nanodelivery systems with peptides show lower cytotoxicity. On the other hand, the systems reduce degradation by enzymes and increase efficiency towards infected cells [32]. Studies have shown that the peptide GIBIM-P5S9K encapsulated in PLGA nanoparticles and evaluated in vitro against bacteria resulted in inhibition of the growth of *E. coli*, *Staphylococcus aureus* (*S. aureus*), and *Pseudomonas aeruginosa* (*P. aeruginosa*) and did not cause haemolysis in the therapeutic concentration range [33]. Tetrahedral framework nucleic acid (tFNA) nanostructures loaded with the antimicrobial peptide GL13K enhanced the inhibition of bacteria and protected the peptide from degradation in the protease-rich extracellular environment [34].

Thirdly, AMPs in a nanodelivery system can permit control of the release rate of peptides and enhance the stability of the peptide in vivo [35,36]. For instance, Zhang and colleagues revealed that, after 4 h oral administration, pexiganan nanoparticles (PNPs) resulted in 10 times higher amounts of PNPs adhered to rat gastric mucosa than free pexiganan [37]. The nanodelivery system was also found to help AMPs in crossing the natural bypass barriers in the organism, including enzymes, digestive liquids in the gastrointestinal tract, and intestinal mucosa, which reduced the first-pass effect and enabled the precise release to the targeted site [38].

Therefore, nanostructures can minimise the undesirable problems of AMPs and can also facilitate their medical uses [39]. Over the last few decades, several types of drug delivery systems have been explored to encapsulate AMPs in preclinical applications for the treatment of infections. Some of these are presented in Table 1, Table 2 and Table 3. These include liposomes [40], micelles [41], dendrimers [42], polymeric nanoparticles [43], liquid crystalline systems [44], hydrogels [45], nanofibres [46], microspheres [47], metal nanocrystalline materials [48], mesoporous silica nanoparticles [49], carbon nanotubes [50], and quantum dots [51]. In this review, the nanodelivery systems applied to AMPs and the mechanisms of transferring them to the disease site will be outlined and discussed.

## 2. Advances in Nanosystems for AMPs Delivery

**Figure 2:**
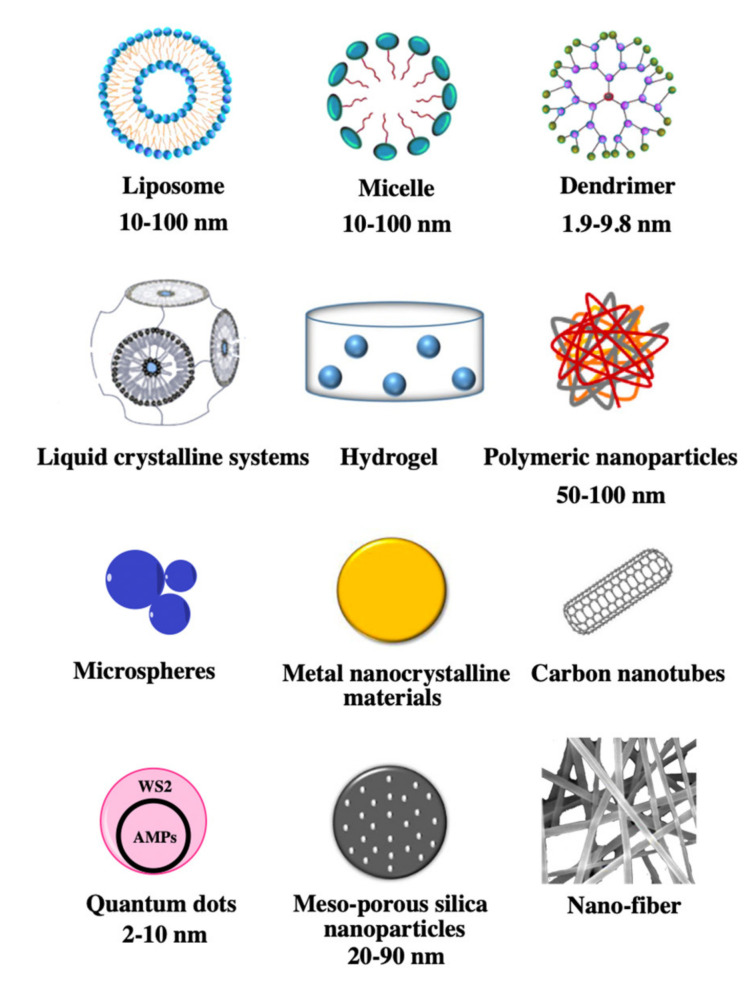
Schematic representation of the 12 nanocarriers used in drug delivery systems.

Currently, research in the use of nanoparticles for AMP delivery has examined many vehicles, including liposomes, micelles, liquid crystalline systems, metal nanocrystalline materials, mesoporous silica nanoparticles, hydrogel, nanofibres, dendrimers, polymeric nanoparticles, carbon nanotubes, and microspheres (Figure 2) [64,65,66,67,68]. Nanocarriers can be classified into several groups according to structures and components. Firstly, liposomes, micelles, and liquid crystalline systems consist of amphiphilic lipid molecules, and they spontaneously arrange as ring-like structures that protect the AMPs from degradation by encapsulating them. Secondly, metal nanocrystalline materials and mesoporous silica nanoparticles can form multipore structures, which can load more AMPs due to their porous structures and associated large surface areas. Thirdly, hydrogel and nanofibres allow AMPs to be equally dispersed in their matrices, which is more suitable for topical administration such as for skin surfaces or eye treatment due to the high degree of dispersion and the controlled release of the drug molecules. Furthermore, dendrimers and polymeric nanoparticles are in a polymeric form, which often prevents degradation by proteases due to their multifaceted structures or shows multifunctionality due to the combination of multiple polymers. Lastly, some nanomaterials tend to have a specific shape: tubular structures include carbon nanotubes, and spherical structures include microspheres.

### 2.1. Liposomes

**Figure 3:**
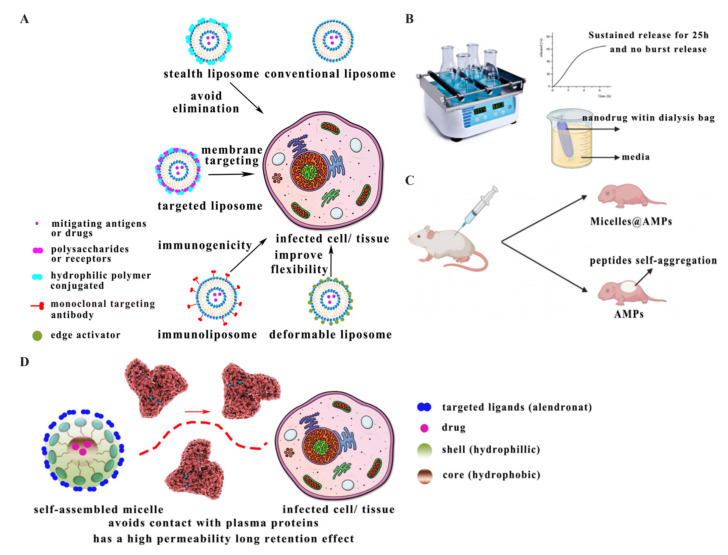
(**A**) Improvement strategies in liposomes adapted from [85]. (**B**) Liposomes with AMPs facilitate a sustained release rate adapted from [40]. (**C**) Micelles with AMPs avoid peptide self-aggregation adapted from [41]. (**D**) Schematic characteristics of micelles.

Liposomes are amphiphilic nanocarriers composed of phospholipids, which load AMPs through trapping hydrophilic molecules in their hydrophilic core or lipophilic drugs in their lipid bilayer [69,70]. Their characteristics are excellent biocompatibility, biodegradability, higher stability, and continued release of encapsulated drugs, so they have many advantages as a vehicle for drug delivery, especially for transdermal drug delivery [35]. However, coupling with liposomes makes the entire drug delivery system immunogenic and more easily causes immune system responses [71]. These nanocarriers are widely used for anticancer, anti-inflammatory, and antibacterial treatments [72]. It was reported that liposomes loaded with AMPs were prepared through the filming–rehydration method, and drug release assay results showed that liposomes could facilitate the sustained release of peptides and facilitate membrane penetration (Figure 3B) [40].

The properties of liposomes depend on phospholipid composition, chemical modification, and surface charge, which are also three important factors to modify liposomes [73]. The conventional liposomes have the limitations of low permeability, low stability, and the possibility of drug leakage. As a result, several improved liposomes were studied, such as stealth liposomes, targeted liposomes, immunoliposomes, and deformable liposomes [74,75] (Figure 3A). The stealth liposomes possess biocompatible hydrophilic polymers (polyethylene glycol or chitosan) covering the surface, which does not cause immunogenicity and reduces the uptake of macrophages [76]. The targeted liposomes have glycoproteins, polysaccharides, or specific receptor ligands on their surface to target specific cells [76]. The immunoliposomes are a class of liposomes that deliver antigens or drugs into tissues and cells [77]. The deformable liposomes are composed of edge activators (sodium cholate, sodium deoxycholate, or Tween-80), which enable the crossing of the stratum corneum to reach viable epidermis flexibly to improve the permeability of traditional liposomal systems [78].

### 2.2. Micelles

Micelles are usually cross-linked, and polymers consisting of micelles self-assemble by chemical bonding [79]. The micelles are characterised by strong permeability and enhanced permeability and retention effects (EPR) [80,81]. Moreover, micelles also have characteristics that encapsulate peptides inside themselves, and their hydrophilic shell avoids peptide contact with plasma proteins (Figure 3D) [82]. The process of loading AMPs is that drugs and polymers with hydrophilic and hydrophobic parts are exposed to a solvent. When the concentration of drug molecules exceeds the critical micelle concentration (CMC), the polar part of the polymers forms the micelle’s outer layer, which faces the solvent. In contrast, the hydrophobic parts face away from the solvent. Finally, they self-assemble into micelles loaded with peptides [83].

It has been shown that micelles loaded with AMPs can reduce cytotoxicity and have potential as therapeutic agents for the treatment of high-density infections. PEG-modified micelles loaded with peptide 73 could reduce the toxicity of peptides and decrease the side effects of peptides self-aggregating in human cells (Figure 3C). Moreover, the result of in vivo activity assays showed that peptide-encapsulated micelles were well absorbed by cells [41]. However, micelle carriers have some drawbacks: they are unsuitable for hydrophilic molecules and unstable in serum, which leads to a low rate of drug release and drug loading [84].

### 2.3. Dendrimers

Dendrimer nanomaterials are polymers with many branches, which consist of three structural components: the core, the branch-like molecules, and the terminal groups [86]. This drug delivery system is characterised by ease of penetrating into the cell membranes due to the active groups on the surface, higher drug loading rates, improved pharmacokinetics, resistance to protein hydrolysis, and a more suitable delivery route to help the drug reach the target site [87,88,89]. It was found that dendrimers loaded with AMPs could be prepared through solid-phase peptide synthesis (SPPS) and purified by HPLC [90]. The dendrimer peptide SB056 was reported to show good antibacterial activity, and the dendrimeric lipopeptides showed strong inhibitory activity against fungi [42]. However, traditional dendrimers also have certain drawbacks: rapid clearance by the immune system and low uptake by cancer cells [91].

**Figure 4:**
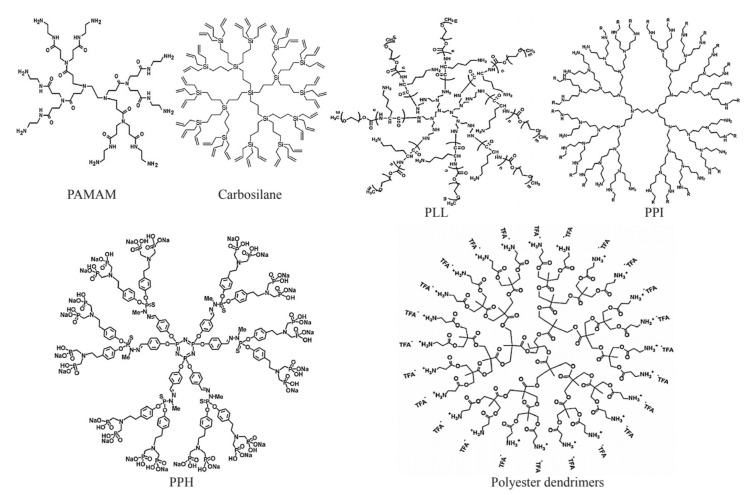
Samples of dendrimers [92,94,98,107,108,109]. PAMAM: PAMAM-G1.0-dendrimer. PPI: PPI-G4 dendrimers. PLL: PEI-g-(PLL-b-PEG). Carbosilane: carbosilane glycodendrimer. PHH: azabisphosphonate-capped dendrimer. Polyester dendrimers: 2,2-bis(methylol)-propionic acid dendrimer.

The most studied dendrimers in the medical field are poly-(amino amide) (PAMAM) [92], poly-(propylene imine) [93,94], poly-(L-lysine) (PLL) [95], carbosilane [96], poly-(phosphor-hydrazone) (PPH) [97], and polyester dendrimers [98] (Figure 4). The first group is the amino-containing dendrimers, which are often cytotoxic and include the following dendrimer types: The PAMAM dendrimers with -NH2 or -OH- terminal groups are mainly studied and have strong polarity and easily modified amine terminations [99]. The PPI dendrimers have multiple tertiary amines in their structures and possess primary amines at the terminus. Besides, PPIs are relatively smaller in size and more hydrophobic than PAMAMs [100]. The PLL dendrimers contain lysine K at the terminus and are more biocompatible [101]. They do not have an internal void space. As a result, this affects the ability of drug loading [102]. The second group is the inorganic dendrimers, which are often hydrophobic. They include the following dendrimer types: The carbosilane dendrimers contain carbon–carbon and carbon–silicon bonds, making the carbosilane flexible, nonpolar, and thermally stable. They can often be modified with polar groups to enhance polarity [103]. The PPH dendrimers have phosphorus atoms in their structure and are nonpolar molecules. They require polar groups to be attached at the periphery of the molecules to make the dendrimers more water-soluble [104]. The polyester dendrimers have good biocompatibility and biodegradability, and the in vitro activity tests showed that these dendrimers have significant antibacterial activity and can act synergistically with bioactive peptides [105,106].

### 2.4. Polymeric Nanoparticles

**Figure 5:**
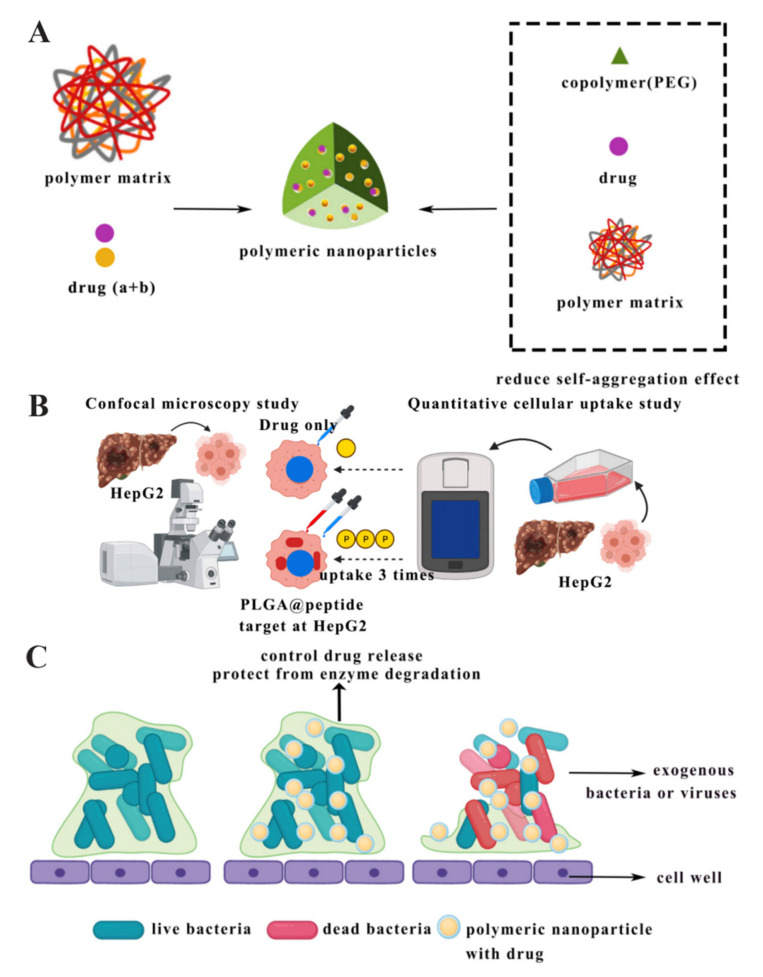
(**A**) Schematic model of polymeric nanoparticles adapted from [115]. (**B**) PLGA nanoparticles with peptides as liver-target drug delivery system adapted from [43]. (**C**) Antibacterial process of polymeric nanoparticles [119].

Currently, the well-studied polymeric nanoparticles are poly-lactic-co-glycolic acid (PLGA), alginate, gelatin, or chitosan polymeric nanoparticles [110]. Polymeric nanoparticles are characterised by the ability to protect the drug from enzymatic degradation, control the rate of drug release, and facilitate drug crossing through the cell wall barrier (Figure 5C) [111,112]. Besides that, another advantage of polymeric nanoparticles is that they can be broken down into biological affinity molecules that can be cleared from the body via metabolic pathways [113]. This drug delivery system could effectively improve drug release methods and rates. On the one hand, there is a liver-targeted drug delivery system, PLGA nanoparticles loaded with FC131 peptide targeted at CXC receptor type 4 (CXCR4) on cancer cells, and in vitro activity results showed that human hepatocellular carcinoma cells (HepG2) took up 3 times more nanoparticles than using the drug alone (Figure 5B) [43]. On the other hand, in regard to the local administration, the release rate of polymeric nanoparticles loaded with peptide exhibited a near-zero or near-first-order distribution and with no burst release, so this could be a promising drug delivery system for the treatment of localised infections [57].

Some successful samples of the polymeric nanoparticle carriers have been widely studied in the laboratory, namely chitosan nanoparticles, PLGA nanoparticles, lipid–polymer hybrid nanoparticles (LPNs), and amorphous nanoparticle complexes (nanocomplexes). The applications of chitosan nanoparticles for mucosal drug delivery and the simultaneous administration of drugs are of interest [114]. It was found that the use of copolymers to prepare chitosan nanoparticles, such as polyethylene glycol (PEG), could reduce their natural tendency to aggregate, making them more biocompatible and stable (Figure 5A) [115]. The PLGA polymeric nanoparticle has the advantages of being biodegradable, biocompatible, and nontoxic. Due to its high drug encapsulation capacity, the PLGA polymeric nanoparticle has been widely used in drug delivery systems [116]. The lipid–polymer hybrid nanoparticles (LPNs) could improve the drug biocompatibility and extend the cycle time in the human body [117]. The amorphous nanoparticle complexes (nanocomplexes) are formed by combining charged polymers with oppositely charged drug molecules [118].

### 2.5. Liquid Crystalline Systems

Liquid crystalline systems (LCSs) are intermediate states between liquid and solid states, and studies on drug delivery of LCSs have mainly been based on using unsaturated monoglycerides to prepare liquid crystals, particularly glycerol monooleate (GMO) [120]. The process of loading AMPs is that the hydrophilic peptides are loaded near the polar head of the liquid crystal structure or in the water channel, the lipophilic peptides are loaded within the lipid bilayer, and the amphiphilic drug is located at the interface [121]. The advantages of this drug delivery system are the capability of controlling drug release, protecting the active ingredient from thermal and photodegradation, increasing the efficiency of loading peptides, and improving bioactivity and adhesion [122,123]. It was shown that LCS has promising applications for dermal drug delivery, and there was an LCS loaded with the AMP D1-23 that not only had greater peptide viscosity and bioadhesion, but also exhibited better activity against *Streptococcus pyogenes* biofilms, showing cumulative effects and no toxicity to human epithelial cells [44].

**Figure 6:**
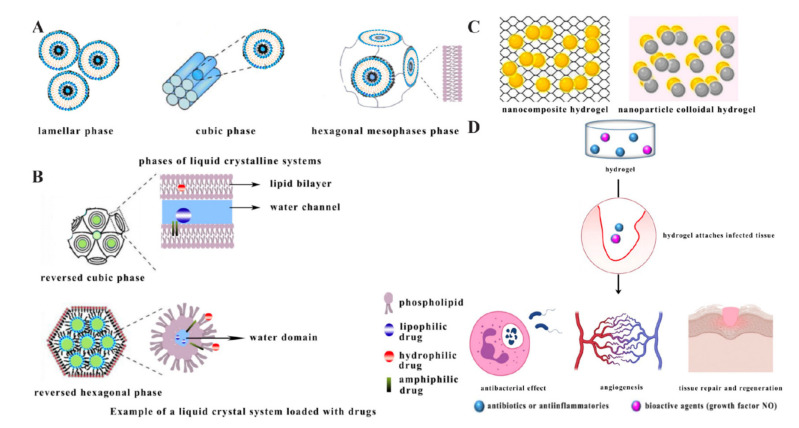
(**A**) Phases of liquid crystal systems adapted from [120]. (**B**) Loading models of Q2 and H2 drug delivery systems adapted from [120]. (**C**) Schematic model of hydrogel adapted from [129]. (**D**) Hydrogel delivers bioactive molecules and is integrative in treating effects.

Liquid crystal systems can be divided into lamellar, hexagonal, and cubic phases depending on the degree of organisation of the molecules (Figure 6A). The lamellar phase is poorly organised and fluid in shape. The cubic phase is the most neatly organised and viscous [120]. The reversed cubic (Q_2_) and the hexagonal mesophases (H_2_) are important samples for drug delivery systems (Figure 6B) [124]. In the Q_2_ model, hydrophilic peptides are located near the polar head of the emulsifier or within the water channel, while lipophilic peptides are located within the lipid bilayer, and amphiphilic peptides are located within the interface [125]. In the H_2_ model, steric conformations consisting of glyceric acid based surfactants such as oleoylglyceric acid (OG) show great potential for drug delivery [126].

### 2.6. Hydrogels

A nanohydrogel is polymer containing three-dimensional lattices. Due to porous three-dimensional structures, hydrogels can absorb aqueous fluids, prolong drug retention, and maintain oxygen penetration when being used for topical administration [127,128]. It was shown that there are two types of hydrogels (Figure 6C) [129]. One is a nanocomposite hydrogel, where nanoparticles are embedded in a hydrogel network. The other is a nanoparticle colloidal hydrogel, where the nanoparticles are used directly as crosslinkers to build the hydrogel network [130]. The pharmacological application of nanohydrogels has become a hot topic in recent years, as they have good biocompatibility and can carry AMPs against skin infections. It was found that a self-assembling octapeptide formed hydrogel model, which was loaded with active peptides and other synergistic drugs when tested for in vitro bioactivity, showed a higher drug retention and provided a combination therapy for topical administration [45]. Notably, hydrogel can be used to deliver bioactive molecules known to accelerate healing (NO); it can promote vascular density and epithelial regeneration, it can fight infection, and finally, it can contribute to skin regeneration (Figure 6D) [131].

### 2.7. Nanofibres

**Figure 7:**
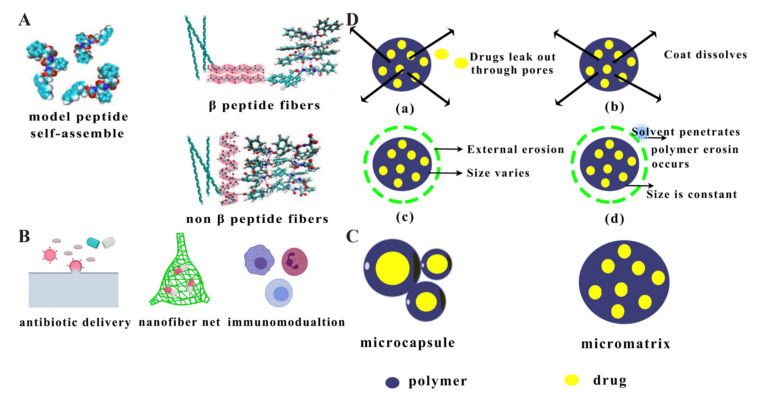
(**A**) Schematic forming model of nanofibres adapted from [136]. (**B**) Different bioactivities of nanofibres. (**C**) Schematic model of microspheres. (**D**) Drug-releasing models of microspheres ((**a**) diffusion, (**b**) dissolution, (**c**) surface erosion, (**d**) overall erosion) adapted from [147].

Nanofibres are thread-like polymers with small sizes ranging from a few microns to a few nanometres. Nanofibres incorporating different therapeutic agents are mainly prepared by the electrospinning method and can load antibiotics, growth factors, plasmid DNA, and AMPs to treat diseases [132]. Studies have shown that self-assembled poly(vinylpyrrolidone)/Eudragit RS100 polymer nanofibres loaded with lysozyme, a special AMP, could inhibit the growth of the oral bacterium *Streptococcus rhamnosus*, and these nanofibres achieved high encapsulation efficiency and prolonged inhibition activity, suggesting that this drug delivery system has excellent potential to deliver therapeutic proteins to the oral mucosa [46]. Notably, nanofibres can form antimicrobial nanonets, and these filamentous networks physically trap nearby bacteria, thereby triggering an antimicrobial and immunomodulating effect by stopping excessive microbial infestation rather than killing the invader directly (Figure 7B) [133,134]. In other words, nanofibres can be used as a potential anticancer therapy method. A previous study showed that one type of self-assembled nanofibre containing amphiphilic peptides and hyaluronic acid could exert an anticancer effect. It provided a potential idea of delivering AMPs to treat cancer in the future [135].

As shown in Figure 7A, self-assembled peptides nanofibre can be classified into β fibres and non-β fibres, according to their different structural characteristics [136]. On the one hand, β-amyloid fibres were proven to have antimicrobial activity against many Gram-positive and Gram-negative bacteria and fungal pathogens, because nanofibres can rapidly form oligomeric states before the bacterial cell wall is perforated [137,138]. On the other hand, there are examples of spiral AMPs forming nanofibres in non-β-amyloid fibre type, which are able to extract phospholipids from bacterial membranes and isolate them in dimer fibrils within hydrophobic regions, preventing their reintegration into the microbial membrane [139].

### 2.8. Microspheres

Microspheres provide the drugs with interspace and allow their dispersal in a polymer matrix of the microspheres. This type of nanocarrier can be divided into three categories: natural polymers, synthetic polymers, and enterosoluble polymers [140]. Microspheres are powders with spherical particles ranging in size from 1 to 1000 μm composed of natural or synthetic polymers that are biodegradable, and their ideal size is less than 200 μm [141]. A previous study showed that microspheres composed of PLGA and chitosan loaded with the AMP KSL-W had an extended antimicrobial effect on oral bacteria and were not cytotoxic to cells, which had therapeutic applications for the treatment of oral infectious diseases [47,139].

As shown in Figure 7C, microspheres can be divided into two categories: microcapsules and micromatrices, depending on how they encapsulate drugs [142]. On the one hand, microcapsules are storage devices in which the drug is encapsulated in a polymeric nanomaterial [143]. On the other hand, micromatrices are those in which the drug is uniformly dispersed in a polymeric matrix [144]. Polymers are divided into natural polymers (ethyl vinyl acetate, proteins, and polysaccharides) and synthetic polymers (PLGA) [145]. Natural polymers are less toxic and biocompatible due to their biodegradability, so they excel in delivering vaccines, proteins, and other therapeutic agents [146]. Synthetic polymers provide relative relief from the possibility of degradation by biologically active enzymes and avoid the production of pathological embolisms in the body [145]. It was reported that drug-releasing models of microspheres can be classified as diffusion, dissolution, surface erosion, and overall erosion. In the diffusion model, peptides can leak out through pores. In the dissolution model, microsphere coatings can dissolve and drugs can then be released, while the erosion models can contribute to external factors and penetration of solvent molecules (Figure 7D) [147].

### 2.9. Metal Nanocrystalline Materials

**Figure 8:**
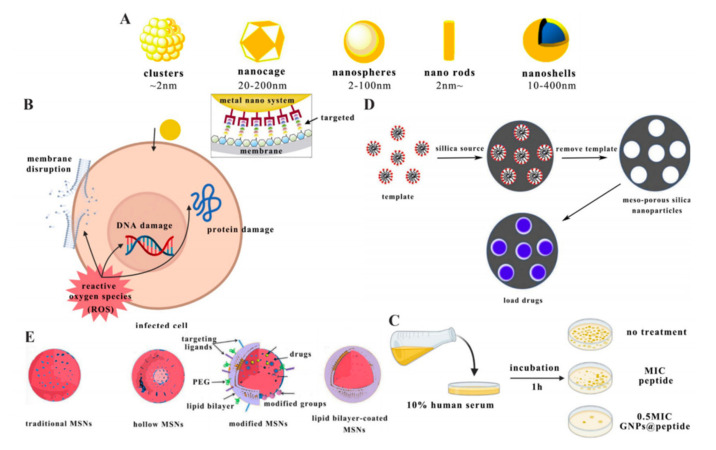
(**A**) Shapes of metal nanocrystalline materials adapted from [155]. (**B**) Antibacterial effect of IONP–peptide adapted from [170]. (**C**) Significant antibacterial effect of GNP–peptide in human serum [149]. (**D**) Schematic drug loading process of mesoporous silica nanoparticles adapted from [165]. (**E**) Schematic models of mesoporous silica nanoparticles adapted from [165].

Metal nanocrystalline nanomaterials have large surface areas, highly ordered pores, and well-defined structures, giving these materials the ability to load and release drugs. In addition, they also have unique features such as ease of synthesis and the possibility to functionalise the surface of the carrier [148,149]. Several metal nanomaterials have specific functions, such as the magnetically responsive superparamagnetic iron oxide nanoparticles (SPIONs) and the photothermally responsive gold nanoparticles (GNPs). GNPs were found to have high antibacterial activity and stability in serum (Figure 8C). They could thus be used for cancer treatment, due to surface plasmon resonance (SPR) theory, which allows them to convert light into heat or scatter energy to kill cancer cells [48,150]. SPIONs have been widely used for targeted drug delivery, and the sizes of these magnetic particles are typically in the range of 10–20 nm. When a magnetic field was applied, the magnetic nanoparticles would collect the magnetic field and did not show magnetism after removal of the magnetic field [151,152]. It was found that the antimicrobial effect and inhibition time of the AMP Ib-M2 were improved through being encapsulated by SPIONs, which had implications for further study for targeted delivery of AMPs [62]. It was also shown that iron oxide nanoparticles (IONPs) could penetrate small capillaries in tissues and integrate into the natural metabolism of the body; they easily contacted with bacterial cells, and their antibacterial mechanism was the production of reactive oxygen, which could cause membrane disruption, protein damage, and DNA damage (Figure 8B) [153,154].

Gold nanocarriers have been studied extensively, and their morphology can be classified into clusters, nanospheres, nanorods, nanoshells, and nanocages depending on their size and toxicity (Figure 8A) [155]. Clusters enhance laser-induced bacterial killing and can also be used for laser treatment in the early stages of cancer [156]. Nanospheres can be used for drug delivery and enhancing Raman imaging [157]. Nanorods can be used for drug delivery, cell imaging, in vivo imaging, and cancer therapy [158]. Nanoshells are larger in size and can be used for in vitro experiments and late-stage cancer therapy [159]. Nanocages can be used for photothermal cancer therapy [160].

### 2.10. Mesoporous Silica Nanoparticles

Mesoporous silica nanoparticles are IUPAC-defined materials with pore sizes between 2 and 50 nm and have a honeycomb porous structure containing silica (SiO_2_) [161,162]. Their advantages are adjustable particle size (50 to 300 nm), uniformly tunable pore size (2–6 nm), high specific surface area, high pore volume, and biocompatibility [163,164]. The drugs are loaded by forming a precarrier from the template reagent and silica source and then removing the template reagent, which provides a rich pore space for the drugs to be loaded in (Figure 8D) [165]. The adjustable particle size and the charge of the particles are essential factors in the composition of intelligent nanocarriers. On the one hand, the adjustable pore size allows different molecular shapes and quantities of drugs to be loaded; the larger the pore size, the greater the release rate. On the other hand, the charge affects the antibacterial capacity [166]. A study tested anionic and cationic mesoporous and nonporous silica particles loaded with AMP (LL-37) and showed that the anionic mesoporous silica particles protected LL-37 from degradation by the associated protease. The nonporous silica particles form a resilient LL-37 surface coating due to their higher negative surface charge, showing particle-mediated membrane interactions and enhancing the antibacterial effect. Positively charged mesoporous silica nanoparticles promoted membrane-disrupting activity but were toxic to human erythrocytes [49].

As shown in Figure 8E, mesoporous silica nanoparticles can be classified into four categories: traditional MSNs, hollow MSNs, lipid bilayer-coated MSNs, and modified MSNs [165]. Hollow MSNs have a hollow core and mesoporous shell structure; the hollow core acts as the storage device, while the mesoporous shell encapsulates the hollow core [167]. The lipid bilayer in lipid bilayer-coated MSNs provides a safe environment for biomolecules, which can eliminate or reduce potential nonspecific adsorption or protein denaturation [168]. Modified MSNs have a high density of surface silanol groups, which can be modified with a variety of organic functional groups. Moreover, the targeting portion of modified MSNs can be modified, which offers the possibility of controlled drug adsorption and release rate and targeted delivery of drugs [169].

### 2.11. Carbon Nanotubes

There as many forms of carbon nanotubes (CNTs), such as hollow spheres, ellipsoids, and tubes [171]. CNTs are formed by rolling graphene sheets into the shape of seamless cylindrical tubes. They can enhance the solubility of drugs and can be used as vaccine individual gene carriers, peptide transporters, NIR photothermal agents, or cancer therapy [172]. Besides that, problems with the BBB, gene delivery, and thermal excision of oncogenic loci can be overcome after chemical or physical functionalisation [173,174,175,176]. Carbon nanotubes tend to aggregate in aqueous media and exhibit potential anti-inflammatory effects but have the problem of high synthetic energy requirements, overdependence on hydrocarbons mainly from petroleum, and low yields [177]. The antibacterial activity of carbon nanotubes against both Gram-positive and Gram-negative bacteria is attributed to the physical bactericidal mode of carbon nanotubes and the induction of oxidative stress leading to cell membrane damage [178]. It has been shown that silver-coated single-walled carbon nanotubes (SWCNTs-Ag) covalently functionalised with TP359 AMPs exhibited additive antimicrobial activity and reduced toxicity and that this strategy would contribute to the development of novel and biologically important nanomaterials [50].

**Figure 9:**
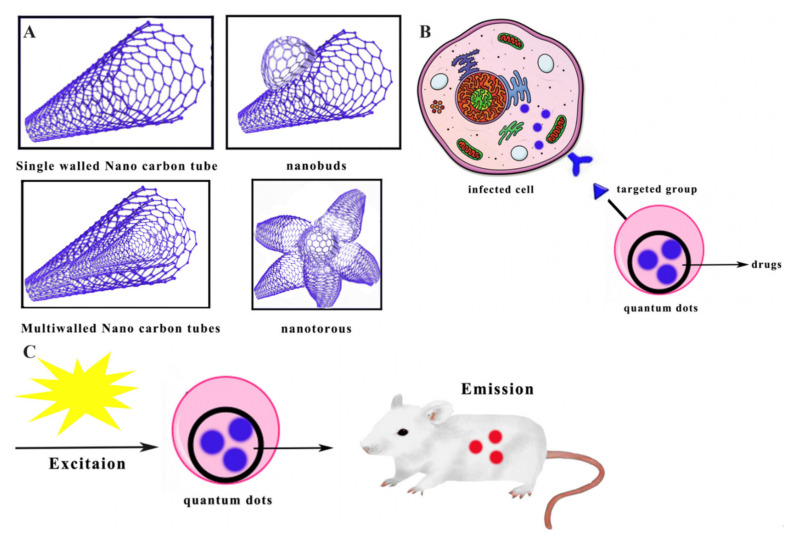
(**A**) Shapes of carbon nanotubes. (**B**) Schematic in vivo targeting for diagnostic or therapeutic purposes of quantum dots, adapted from [182]. (**C**) Schematic bioluminescence imaging process of quantum dots, adapted from [63].

As shown in Figure 9A, carbon nanotubes can be classified as single-walled carbon nanotubes, multiwalled carbon nanotubes, nanotorous structures, and nanobuds [179]. Single-walled nanotubes (SWNT) are close to 1 nm in diameter, while their length may be different. Multiwalled carbon nanotubes are structurally composed of multiple layers of graphite, which are rolled in layers to form a tubular structure that optimises the solubility and dispersion of single-walled carbon nanotubes [180]. Nanobuds consist of fullerenes covalently bonded to the sidewalls of carbon nanotubes [181]. Nanotorous structures are single-walled carbon nanotorous structures with tapered tips, which have a high specific surface area and excellent electrical properties and are often used as electrode materials [182].

### 2.12. Quantum Dots

Quantum dots are fluorescent semiconductor nanocarriers, typically consisting of hundreds to thousands of group II and VI molecular atoms, with unique photophysical properties. As shown in Figure 9B, the carriers are characterised by their ability to be used as imaging of biological systems, including in vitro imaging of immobilised cells and in vivo targeting for diagnostic or therapeutic purposes [183]. Most quantum dots consist of three parts: the core, the shell, and the capping material [184]. The key factor in the antimicrobial performance of quantum dots is thought to be reactive oxygen species (ROS), which target cell wall and membrane components such as lipoprotein acids and phosphatidylglycerols [185]. It has been shown that tungsten disulphide (WS_2_) quantum dot embedded AMPs KG18 and VR18 enhanced the antimicrobial and antibiofilm abilities of these peptides due to multiple condensation reactions of the embedding peptides, which could be used as antimicrobial agents. Besides that, they could be used as selective pathogen imaging agents due to the property of fluorescence, light stability, and small size (Figure 9C) [63].

## 3. Features and Applications for Constructing Nanodelivery Systems Loaded with AMPs

**Figure 10:**
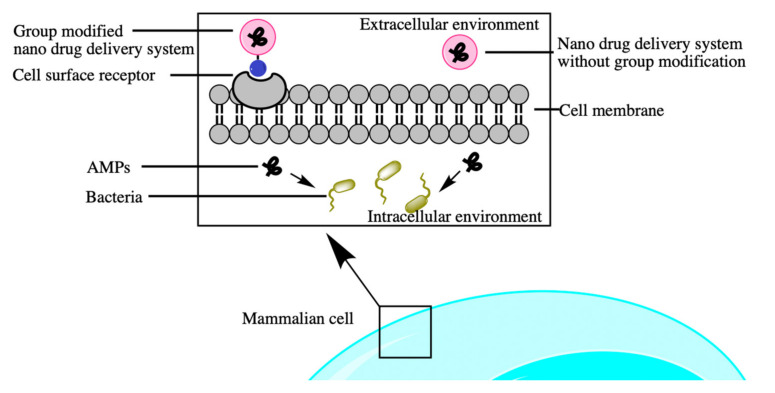
Two systems of delivering AMPs to infected cells.

Nanotechnology has two main approaches to construct drug delivery systems, namely modified nanodrug delivery systems and nanodrug delivery systems without chemical modification (Figure 10) [186]. On the one hand, nanodrug delivery systems without modification are also called physical modification systems and allow AMPs to be adsorbed into nanocarriers to produce nondirectional and passive drug delivery. These systems load different molecular sizes by controlling the nanocarriers’ size and shape [187]. A study has shown that monolaurin–lipid nanocapsules (ML-LNCs) loaded with the AMPs AP114 and AP138 had an inhibition effect on methicillin-resistant *S. aureus* (MRSA). This synergistic dosing method resulted in lower use of drugs, lower risk of toxicity, and lower probability of drug resistance [30].

On the other hand, modified nanodrug delivery systems are also called chemical modification systems, which have become known as targeted drug delivery. Here, surfaces are modified with ligands or other components to allow the nanocarrier to interact with the intended site on the human cells or to fluoresce them for easy detection [188,189]. It was reported that the moieties or markers were used to modify nanocarriers and provide ideas for targeted drug delivery therapies. Besides that, it was found that PLGA nanoparticles loaded with peptide and fluorescent markers (BSA-FITC) affected the process of intracellular drug absorption and increased the extent of endocytosis. When fluorescent markers were combined with peptides for therapeutic use, they could be used in such a way to increase the uptake of the peptide and facilitate the penetration of the marker into the cell, offering new ideas for site-specific treatments [190].

Many ligands have been reported for chemical modification systems. One is the targeting ligand Histatin 5, which is both a targeting ligand for binding to the Ssa1/2p receptor on the fungal cell wall and an antifungal molecule that targets *Candida albicans* (the major systemic fungal pathogen in humans) in concert with antibiotics [191]. The other is the sodium cholate molecule, which can play a synergistic role with AMPs. When they penetrate into the lung surfactant model, peptides electrostatically interact with the lipid polar head of the cell wall and can enhance the antibacterial effect of noncharged AMPs towards the negative-charged bacterial membrane. The use of sodium cholate ligands to form nanoparticles with AMPs allows the sodium cholate molecules to diffuse around the lipid polar head and protect the peptides from interaction with the lipid polar head, allowing them to be freely delivered to the aqueous phase. This is a good system for delivering drugs to the lung [192].

In general, it is worth noting that these two nanodrug delivery systems, whether physical or chemical modification systems, have the following common characteristics: One is they can enter cells through enhanced permeability and retention effect (EPR) [193]. The other is that they can be pH-responsive [194], temperature-responsive [195], actively targeted by target receptor response [196], redox-sensitive [197], enzyme-responsive [198], magnetic field-responsive [199], and light-responsive [200].

**Figure 11:**
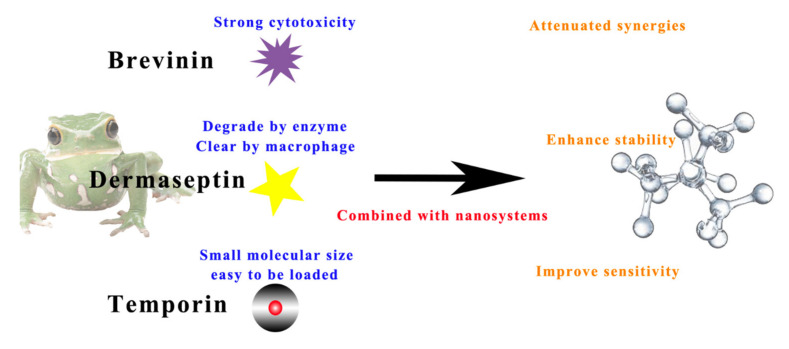
Application samples for constructing nanodelivery systems with AMPs.

The combination of active peptides with nanosystems to treat human diseases has three main purposes (Figure 11). Firstly, the strong haemolytic side effects of peptides could be reduced by combining with nanodelivery technology, useful for AMPs such as members of the brevinin family. According to the database of anuran and defence peptides (DADP), more than 350 brevinins have been identified in the two main subfamilies, brevinin 1 and brevinin 2 [201]. Almost all brevinin superfamilies have high antibacterial activity against Gram-positive and Gram-negative bacteria and fungal pathogens. However, they are limited by their potent haemolytic properties, so their chemical modification or combination with nanodelivery systems to achieve a reduction in toxicity is necessary for the development of AMPs [202].

Secondly, dermaseptin peptides are cationic and amphiphilic, which relate to cell membrane interactions; these peptides are not selective for tumours and may damage healthy cells. In addition, they have the possibility of being hydrolysed by peptidases. Therefore, it is important to combine with nanoparticle systems to overcome the nonspecific cytolytic effect, transport to target cells, and protect them from enzymatic degradation and macrophage clearance [203,204]. There was one study that prepared DStomo01–chitosan nanoparticles and encapsulated the drug and controlled its release rate, overcoming its susceptibility to enzymatic degradation [205].

Lastly, temporin peptides have been used as antimicrobial agents and have better potential to combine with nanomaterials as they are of low molecular size and hydrophobicity and have significant antimicrobial activity, structural chemotaxis, and histamine-promoting properties [206]. Some temporins are effective against a wide range of pathogens, and their antibacterial mechanism is based on cytoplasmic membrane perturbation [207]. Besides this, some peptides also have chemotactic and immunomodulatory effects, such as temporin B and temporin L, which can penetrate lipid monolayers [208]. From a therapeutic point of view, temporin, as a low-molecular-weight active drug component, can be better loaded into nanocarriers and perform bioactivities better. Some nanosubstrates with microstructures can load AMPs to form nanodelivery systems with optimal pharmacokinetics; thus, they improve the efficacy and toxicological safety of the treatment [209]. There was a study that showed that a temporin A polymer was prepared and that it achieved an antimicrobial effect through cytoplasmic membrane perturbation. The synthesised polyester matrix can be used as a potential application for long-term controlled delivery of AMPs for local infections [210]. It has also been shown that temporin B–chitosan nanoparticles with an encapsulation rate of up to 75%, could significantly reduce the cytotoxicity of the peptide to mammalian cells and prolong antimicrobial effects [59].

## 4. Conclusions and Challenges in Nanotechnology for AMP Delivery

Nanotechnology combined with bioactive peptides, however, has also encountered some challenges. Firstly, although the application of nanotechnology in combination with marketed peptides is a promising approach, the issue of huge cost needs further consideration. The United States Food and Drug Administration (FDA) approved 48 peptide drugs, of which eight were peptides not encapsulated in nanomaterials [211]. The production cost of synthetic peptides was estimated to be USD 300–500 per gram. This limits their applications. The inclusion of nanomaterials incurs additional production costs. As a result, it is sometimes not possible to predict the economic benefits from nanotechnology commercialisation [212,213,214]. Secondly, there are some problems in preparing nanodelivery systems containing peptides, because the exposure of the peptide to organic solvents and external forces such as ultrasound can alter the morphology and activity of the peptide [215]. Temperature is also a sensitive parameter when preparing nanodelivery systems containing peptides. During the synthesis process, high temperature or extremely low temperature may affect the ion pairing between the nanocarrier and the peptide, thereby affecting the stability of the loaded peptide and ultimately leading to the denaturation of the peptide [216]. Thirdly, the main obstacle is that their safety and cytotoxicity must be further evaluated before treating human diseases [217]. There is still a lot of uncertainty in nanodrug delivery systems and a lack of examples in clinical treatment.

In conclusion, based on the above discussion, there are several suggestions for choosing the appropriate nanodelivery system for AMPs. Firstly, in terms of some strongly cytotoxic AMPs, liposomes, polymeric nanoparticles, and microspheres can be chosen for toxicity reduction. Secondly, in terms of AMPs being used for local infections, micelles, liquid crystalline systems, hydrogels, and nanofibres are more suitable for epithelial or ocular administration, which could enhance the permeability and retention of AMPs. On the other hand, dendritic polymers and mesoporous silica nanoparticles could protect AMPs from enzyme hydrolysis to enhance their bioactivities. In regard to helping AMPs target human cells or exert other functional responses, polymeric nanoparticles, metal nanocrystalline materials, and quantum dots could provide more ideas for modifying or designing novel peptide delivery systems.

However, nanocarriers still have potential as active peptide delivery systems. They offer many advantages such as protection against extracellular degradation, targeted therapy, and improved pharmacokinetic properties [218]. In the clinical field, nanocarriers are still under study for encapsulating AMPs as therapeutic and immunomodulatory agents [39]. Not only can nanocarriers help to deliver AMPs, but also AMPs can be designed as nanoform vectors for loading other drugs [219]. The combination of nanocarriers and peptides can inhibit the growth of bacteria strains that are resistant to last-resort antibiotics and achieve the purpose of treating infections. Nickel-doped zinc oxide combined with black phosphorus nanocomposite with active peptide polymyxin B could be used against *E. coli* resistant to last-resort antibiotic polymyxin [220]. As AMPs are becoming more widely and intensively researched, more investments and further studies about combining peptides with nanodelivery systems are likely to be a future study hotspot in medicine.

## Figures and Tables

**Table 1:** An overview of liposomes, micelles, and dendrimers as nanodrug delivery systems for AMPs and their potential applications for treatment of infections.

System	Liposome		Micelle		Dendrimer			
			Phospholipid Micelles	PLGA-PEG Micelles	PLL	PAMAM	PPI	Carbosilane
**Delivery mechanism**	Passive delivery	Passive delivery	Passive delivery	Passive delivery	Passive delivery	Passive delivery	Targeted delivery	Passive delivery
**Peptide**	Synthetic peptide	Alyteserin-1c	Peptide 73	HnMc	G3KL	SB056	SB105	AMP3^1^
**Target**	Methicillin-resistant *Staphylococcus aureus (MRSA)*	*Listeria monocytogenes, E. coli*	MRSA	*S. aureus,* *P. aeruginosa, E. coli*	*A. baumannii, P. aeruginosa*	*Enterococcus faecalis, Staphylococcus epidermidis, S. aureus*	HPV infection of 293TT cells	*E. coli, S. aureus*
**Potential application**	Bacterial infection	Bacterial infection	High-density infections	Bacterial infection	Bacterial infection	Bacterial infection	HPV infection	Bacterial infection
**Reference**	[40]	[52]	[41]	[53]	[54,55]	[42]	[54]	[56]

Abbreviations: PPI: poly-(propylene imine); PLL: poly-(L-lysine); PAMAM: poly-(amino amide); AMP3: H-CRKWVWWRNR-NH_2_.

**Table 2:** An overview of AMPs contained in nanodrug delivery systems composed of polymeric nanoparticles, liquid crystalline systems, and mesoporous silica nanoparticles and their potential applications.

System	Polymeric Nanoparticle			Liquid Crystalline System		Hydrogel	Mesoporous Silica Nanoparticle
	LPN	PLGA	Chitosan	Cubic Phase	Hexagonal Mesophases		
**Delivery mechanism**	Passive delivery	Passive delivery	Passive delivery	Passive delivery	Passive delivery	Passive delivery	Passive delivery
**Peptide**	Citropin 1.1	GAM019	Temporin B	D1–23	AP114, DPK-060, and LL-37	Lysozyme	LL-37
**Target**	MRSA	MRSA, *E. coli*	*S. epidermidis*	*Streptococcus mutans*	MRSA, *E. coli*	*Streptococcus ratti*	*E. coli*
**Potential application**	Bacterial infection	Bacterial infection	Bacterial infection	Bacterial infection	Bacterial infection	Oral infection	Bacterial infection
**Reference**	[57]	[58]	[59]	[44]	[60]	[46]	[49]

Abbreviations: PLGA: poly-(lactic-co-glycolic acid); LPN: lipid–polymer hybrid nanoparticle.

**Table 3:** An overview of AMPs contained in nanodrug delivery systems composed of microspheres, metal nanocrystalline materials, carbon nanotubes, and quantum dots and their potential applications.

System	Microsphere		Metal Nanocrystalline Material		Carbon Nanotube	Quantum Dot
	PLGA/Chitosan (Micromatrix)	Alginate/Chitosan (Microcapsule)	Gold Nanoparticles	SPIONs		WS_2_
**Delivery Mechanism**	Passive delivery	Passive delivery	Passive delivery	Passive delivery	Passive delivery	Targeted delivery
**Peptide**	KSL-W	Dermicidin-1-L	Cecropin-melittin	Ib-M2	APs	KG18 and VR18
**Target**	*Fusobacterium nucleatum*	*S. aureus, Klebsiella pneumoniae*	*S. aureus, E. coli*	*E. coli*	*Streptococcus pyogenes, E. coli*	*P. aeruginosa, Candida albicans*
**Potential application**	Oral infection	Bacterial infection	Bacterial infection	Bacterial infection	Bacterial infection	Antimicrobial therapy and bioimaging.
**Reference**	[47]	[61]	[48]	[62]	[50]	[63]

Abbreviations: PLGA: poly-(lactic-co-glycolic acid); SPIONs: superparamagnetic iron oxide nanoparticles.

## Data Availability

Not applicable.
